# Novel human microbe-disease association prediction using network consistency projection

**DOI:** 10.1186/s12859-017-1968-2

**Published:** 2017-12-28

**Authors:** Wenzheng Bao, Zhichao Jiang, De-Shuang Huang

**Affiliations:** Institute of Machine Learning and Systems Biology, School of Electronics and Information Engineering, Tongji University, Caoan Road 4800, Shanghai, 201804 China

**Keywords:** Microbe, Disease, Association prediction, Network consistency projection

## Abstract

**Background:**

Accumulating biological and clinical reports have indicated that imbalance of microbial community is closely associated with occurrence and development of various complex human diseases. Identifying potential microbe-disease associations, which could provide better understanding of disease pathology and further boost disease diagnostic and prognostic, has attracted more and more attention. However, hardly any computational models have been developed for large scale microbe-disease association prediction.

**Results:**

In this article, based on the assumption that microbes with similar functions tend to share similar association or non-association patterns with similar diseases and vice versa, we proposed the model of Network Consistency Projection for Human Microbe-Disease Association prediction (NCPHMDA) by integrating known microbe-disease associations and Gaussian interaction profile kernel similarity for microbes and diseases. NCPHMDA yielded outstanding AUCs of 0.9039, 0.7953 and average AUC of 0.8918 in global leave-one-out cross validation, local leave-one-out cross validation and 5-fold cross validation, respectively. Furthermore, colon cancer, asthma and type 2 diabetes were taken as independent case studies, where 9, 9 and 8 out of the top 10 predicted microbes were successfully confirmed by recent published clinical literature.

**Conclusion:**

NCPHMDA is a non-parametric universal network-based method which can simultaneously predict associated microbes for investigated diseases but does not require negative samples. It is anticipated that NCPHMDA would become an effective biological resource for clinical experimental guidance.

**Electronic supplementary material:**

The online version of this article (10.1186/s12859-017-1968-2) contains supplementary material, which is available to authorized users.

## Background

In the past few decades, accumulating evidence has demonstrated that human lives strongly rely on a diverse, complex and dynamic microbial community, including bacteria, protozoa, viruses, eukaryotes, archea and so on [1]. Tremendous microorganisms inhabit a range of human organs such as skin, gut, mouth, stomach and vagina, where a commensal relationship between microbe and human host has been established after a long term adaptive co-evolution. Recently, more and more reports have confirmed that microbiome could benefit human health by maintaining normal homeostasis, strengthening immune system, promoting host’s metabolism, and modulating development of gastrointestinal tract [2]. Typically, it is reported that the number of bacterial cells in an adult intestine reaches 10^14^, which is approximately 10 times as the number of total human cells [3]. More than 5,000,000 genes (outnumbering the human genetic potential by two orders of magnitude) are contained in the combined genomes of these bacteria, and tens of trillions of gene products are involved in a variety of biochemical and metabolic activities, providing important complement to host physiology [1, 4]. In a sense, it is reasonable to regard gut bacteria as an additional ‘organ’ for its equal metabolic capacity as the liver [5]. Essential gut bacteria could effectively promote nutrient absorption by assisting decomposition of indigestible polysaccharides and production of indispensable vitamins [3]. Furthermore, they provide important protection against invasion of food-borne pathogens by impacting on proliferation and differentiation of host intestinal epithelium [6, 7]. However, a system understanding of how these biochemical activities achieve still remain largely unknown.

According to recent reviews, microbiota in human bodies could be significantly influenced by both maternal genetics [8, 9] and environment variables including hygiene of food and residence [3], change of season [10], usage of antibiotics [11] and personal diet of host [12, 13]. These pivotal factors interact with each other and build a dynamic relationship system, modifications of which would lead to imbalance of microbial community and further impact on transcriptomic, proteomic and metabolic profiles of related microorganisms. With the rapid development of high-throughput sequencing techniques as well as newly developed computational tools, accumulating evidence has demonstrated that disorders of host’s microbiota would increase the incidences of various complex human diseases such as liver diseases [14], diabetes [15], asthma [16], infectious colitis [17] and even cancers [18, 19]. For example, to identify action of microorganisms in asthmatic airways, Hilty et al. [20] studied 24 adult subjects composed of 11 patients with asthma, 5 patients with chronic obstructive pulmonary disease (COPD) and 8 healthy individuals, and found that adult asthma and COPD was inextricably related to high abundance of *Proteobacteria* and *Haemophilus* as well as low abundance of *Bacteroidetes* and *Prevotella.* Mondot et al. [21] analyzed DNA sequences extracted from fecal samples which are collected from 16 Crohn’s disease (CD) patients and 16 healthy subjects. As a result, they observed decrease of *Faecalibacterium prausnitzii* abundance as well as increase of *Escherichia coli* abundance in CD-patients’ fecal samples compared with the controls’. In addition, Chen et al. [22] discovered a shift in composition of liver microbiota when comparing healthy and liver cirrhosis samples. In this study, liver cirrhosis was observed to be related with increase in the abundance of *Bacilli*, *Enterobacteriaceae*, *Fusobacteriaceae*, *Pasteurellaceae*, *Proteobacteria*, *Streptococcaceae* and *Veillonellaceae* as well as decrease in the abundance of *Bacteroidaceae* and *Lachnospiraceae*.

As mentioned above, identifying potential associations between microbes and diseases has a long-term theoretical and practical significance not only for better understanding of disease formation and development mechanisms but also for discovery of novel medical solutions for disease prevention, diagnosis, treatment and prognosis [23]. However, current amount and quality of known microbe-disease associations are far from satisfying the requirements of medical research. In traditional way, researchers attempt to obtain new associations between microbes and diseases by biological or clinical experiments, which demand a large quantity of time and cost. With the rapid development of computer technology, more and more computational models have been developed to predict potential miRNA-disease associations [24, 25], potential lncRNA-disease associations [26] and potential drug-target interactions [27], where machine learning-based and similarity measure-based models have shown their outstanding prediction ability. It is essential to logically extend these prediction methods into microbe-disease association prediction field. Recently, Ma et al. [23] manually collected experimentally verified microbe-disease associations from published clinical research reports and built the first Human Microbe-Disease Association Database (HMDAD). Based on the records from HMDAD, powerful computational models could be developed to prioritize candidate microbes for investigated diseases in large-scale.

In this paper, based on the assumption that microbes with similar functions tend to share similar association or non-association patterns with similar diseases, we developed the model of Network Consistency Projection for Human Microbe-Disease Association prediction (NCPHMDA) to uncover potential microbe-disease associations. By taking advantages of known microbe-disease association network and Gaussian interaction profile kernel similarity network for microbes and diseases, NCPHMDA achieved reliable prediction performance. NCPHMDA could be applied to new microbes without any known associated diseases as well as new diseases without any known associated microbes. As a non-parametric network-based prediction method, NPCHMDA demonstrated obvious advantages when the known experimentally verified microbe-disease associations are insufficient. Three validation frameworks, global leave-one-out cross validation (global LOOCV), local leave-one-out cross validation (local LOOCV) and 5-fold cross validation (5-fold CV), have been implemented to evaluate the performance of NCPHMDA. As a result, NCPHMDA achieved AUCs of 0.9093, 0.7953, and 0.8918 in global LOOCV, local LOOCV, and 5-fold CV, respectively. Moreover, colon cancer, asthma and type 2 diabetes were taken as three independent case studies, where 9, 9, 8 out of top 10 predicted microbes were successfully confirmed by recent experimental and clinical reports, respectively.

## Methods

### Human microbe-disease associations

Human Microbe-Disease Association Database (HMDAD, http://www.cuilab.cn/hmdad) [23] integrated 483 high-quality microbe-disease entries, which were mainly collected from 16S RNA sequencing–based microbial literature. After removing the duplicate association records, 450 distinct microbe-disease associations were finally obtained, including 292 microbes and 39 diseases. Adjacency matrix *A* was adopted to quantify the relationship between diseases and microbes, where binary element *A(i,j)* denotes the presence or absence of association between disease *d(i)* and microbe *m(j)* (‘0’ represents absence while ‘1’ represents presence). Furthermore, to represent the number of microbes and diseases investigated in this article, variables *nm* and *nd* are respectively defined.

### Gaussian interaction profile kernel similarity for diseases

Gaussian interaction profile kernel similarity for diseases was calculated based on the assumption that diseases with similar phenotypes always share similar association and non-association pattern with functionally similar microbes. We defined binary vector *IP(d(i))* to denote the interaction profile of disease *d(i)*, which could be obtained by observing whether *d(i)* has known association with each microbe or not (i.e. the *ith* row of adjacency matrix *A*). Then, Gaussian interaction profile kernel similarity matrix *KD* could be constructed after calculation of similarity value between each disease pair.

1 $$ KD\left(d(i),\kern0.5em d(j)\right)\kern0.5em =\kern0.5em \exp \kern0.3em \left(-{\gamma}_d{\left\Vert IP\left(d(i)\right)\kern0.3em -\kern0.3em IP\left(d(j)\right)\right\Vert}^2\right) $$

2 $$ {\gamma}_d=\gamma {\hbox{'}}_d/\left(\frac{1}{nd}\sum \limits_{i=1}^{nd}{\left\Vert IP\left(d(i)\right)\right\Vert}^2\right) $$

where value of parameter *γ*_*d*_ controls the bandwidth of Gaussian kernel. As presented in eq. (2), *γ*_*d*_ could be further calculated by dividing a new bandwidth parameter *γ* ^*’*^_*d*_ by average number of associations with microbes for all the diseases. Here, we set *γ* ^*’*^_*d*_ = 1 according to previous studies [28].

### Gaussian interaction profile kernel similarity for microbes

Adopting the same approach, Gaussian interaction profile kernel similarity between microbe *m(i)* and *m(j)* could be obtained as follows.

3 $$ KM\left(m(i),\kern0.5em m(j)\right)\kern0.5em =\kern0.5em \exp \kern0.3em \left(-{\gamma}_m{\left\Vert IP\left(m(i)\right)\kern0.3em -\kern0.3em IP\left(m(j)\right)\right\Vert}^2\right) $$

4 $$ {\gamma}_m=\gamma {\hbox{'}}_m/\left(\frac{1}{nm}\sum \limits_{i=1}^{nm}\kern0.1em {\left\Vert IP\left(m(i)\right)\right\Vert}^2\right) $$

where *IP(m(i))* represents the interaction profile of microbe *m(i)* (i.e. the *ith* column of adjacency matrix *A*). Normalized kernel bandwidth parameter *γ*_*m*_ could be calculated in the similar way as *γ*_*d*_, where we select *γ* ^*’*^_*m*_ = 1 according to Van et al. [28].

### NCPHMDA

**Fig. 1:**
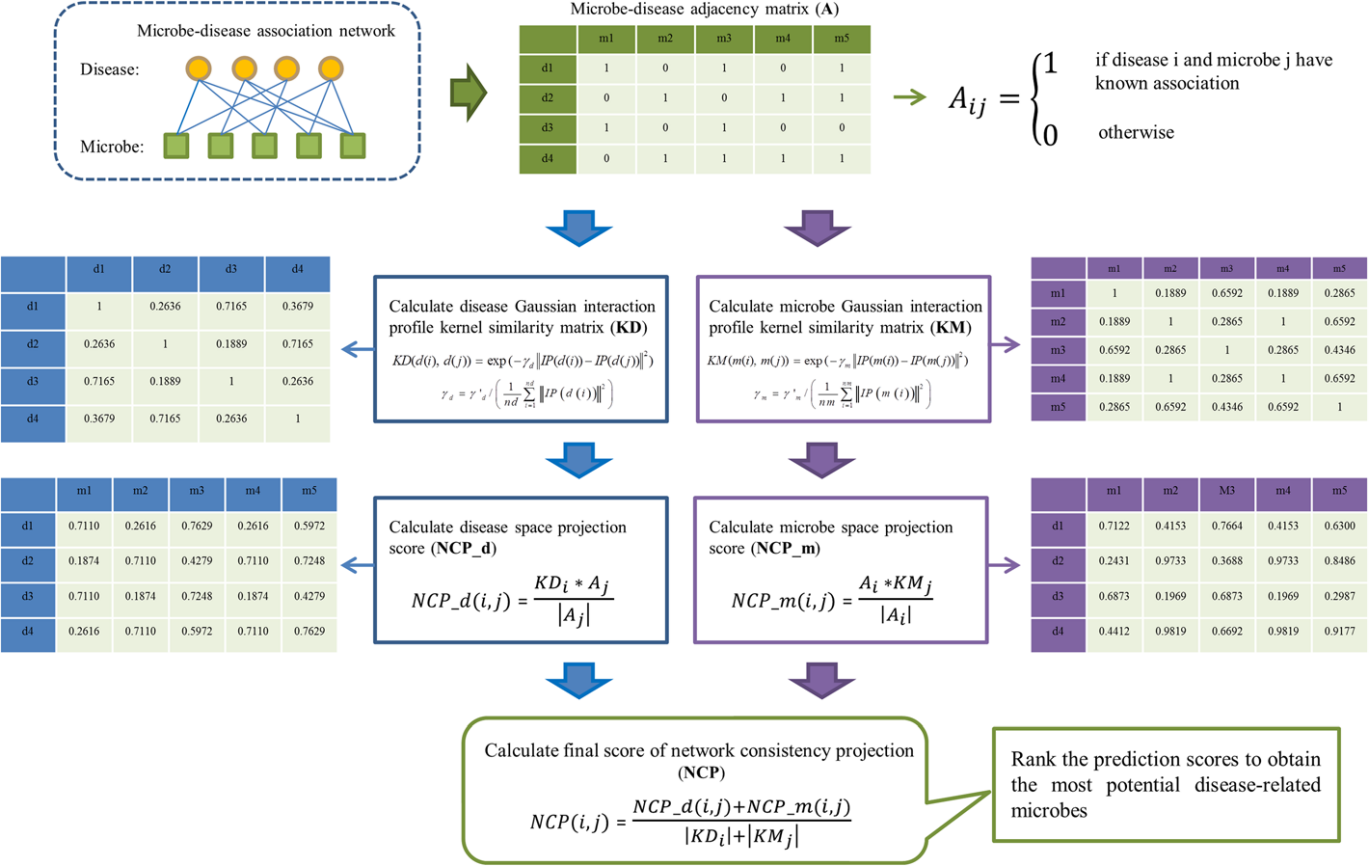
Flowchart of NCPHMDA demonstrating the basic ideas of predicting potential microbe-disease associations by integrating known microbe-disease associations and Gaussian interaction profile kernel similarity for microbes and diseases

As shown in Fig. 1, NCPHMDA is a network-based prediction model which measures the relevance between microbes and diseases by calculating the nodes’ similarity in heterogeneous networks. Here, heterogeneous networks consist of microbe-disease association network constructed based on records from HMDAD [23] database, Gaussian interaction profile kernel similarity for diseases, and Gaussian interaction profile kernel similarity for microbes.

NCPHMDA first calculates two network consistency projection scores, disease space projection score and microbe space projection score, separately. The disease space projection score is calculated as follows.

5 $$ NCP\_d\left(\mathrm{i},\mathrm{j}\right)=\frac{{KD_i}^{\ast }{A}_j}{\left|{A}_j\right|} $$

where *KD*_*i*_ is the *ith* row of matrix *KD* and the vector represents the similarities between disease *i* and all other diseases. *A*_*j*_ is the *jth* column of matrix *A* and the vector represents the associations of microbe *j* and all diseases. |*A*_*j*_| is the norm of vector *A*_*j*_. Matrix *NCP_d* is the projection score of disease Gaussian interaction profile kernel similarity network (represented as matrix *KD*) on the known microbe-disease association network (represented as matrix *A*), where the element *NCP_d(i,j)* in row *i* and column *j* is the network projection of *KD*_*i*_ and *A*_*j*_. Notably, the more similar diseases and disease *i* are, the more diseases associated with microbe *j*, and the smaller angle between *KD*_*i*_ and *A*_*j*_, the greater network consistency projection score *NCP_d(i,j)* is. The microbe space projection score could be combined and normalized in the similar way as follows.

6 $$ NCP\_m\left(\mathrm{i},\mathrm{j}\right)=\frac{{A_i}^{\ast }{KM}_j}{\left|{A}_i\right|} $$

where *A*_*i*_ is the *ith* row of matrix *A*, which consists of associations of disease *i* and all microbes. *KM*_*j*_ is the *jth* column of matrix *KM*, which comprises the similarities of microbe *j* and all other microbes. Matrix *NCP_m* is the projection score of microbe Gaussian interaction profile kernel similarity network (represented as matrix *KM*) on the known microbe-disease association network (represented as matrix *A*), where the element *NCP_m(i,j)* in row *i* and column *j* is the network projection of *KM*_*j*_ and *A*_*i*_. Remarkably, the more similar microbes and microbe *j* are, the more microbes associated with disease *i*, and the smaller angle between *KM*_*j*_ and *A*_*i*_, the greater network consistency projection score *NCP_m(i,j)* is.

Finally, we could combine and normalize *NCP_d* and *NCP_m* as follows.

7 $$ NCP\left(\mathrm{i},\mathrm{j}\right)=\frac{NCP\_\mathrm{d}\left(\mathrm{i},\mathrm{j}\right)+ NCP\_m\left(\mathrm{i},\mathrm{j}\right)}{\left|{KD}_{\mathrm{i}}\right|+\left|{KM}_{\mathrm{j}}\right|} $$

where *NCP_d(i,j)* and *NCP_m(i,j)* are the projection scores in disease space and microbe space of disease *i* and microbe *j*, respectively. *KD*_i_ is the *ith* row of matrix *KD*, *KM*_*j*_ is the *jth* column of matrix *KM*, and |·| is the normalization operation. *NCP* is the final score matrix of network consistency projection, which measures the association probability between each microbe-disease pair.

## Results and discussion

### Performance evaluation

We implemented LOOCV and 5-fold CV on the experimentally verified microbe-disease associations recorded in HMDAD database to evaluate the prediction performance of NCPHMDA. In validation frameworks of LOOCV, we left out each known microbe-disease association in turn for model testing while adopted other known microbe-disease associations as training samples. According to whether all the diseases were investigated simultaneously or not, LOOCV could be further split into global LOOCV and local LOOCV. When global LOOCV was implemented, all the microbe-disease pairs without known supporting evidence in HMDAD were adopted as candidate samples, while when local LOOCV was implemented, we only took microbes without known confirmed relevance with investigated disease as candidate samples. In the framework of 5-fold CV, we randomly divided all the known microbe-disease associations into 5 average groups, 4 of which were used as training samples for model learning and the remaining one was used as testing samples for model evaluation. It needs to be emphasized that we repeated 5-fold CV for 100 times to reduce the potential deviations caused by random sample divisions. Each testing sample was ranked with all candidate samples, where the model was considered to achieve a successful prediction if the rank of the testing sample exceeds the given threshold. After setting a series of thresholds, corresponding true positive rates (TPR, sensitivity) were calculated by counting percentages of the test samples with higher ranks than investigated thresholds. Meanwhile, false positive rates (FPR, 1-specificity), which denote the percentages of the negative samples exceeding the given thresholds, were also obtained. To visualize the prediction ability, receiver-operating characteristics (ROC) curves were then drawn by plotting TPR against FPR at different thresholds. Area under ROC curve (AUC) was finally calculated as an essential performance evaluation criterion.

**Fig. 2:**
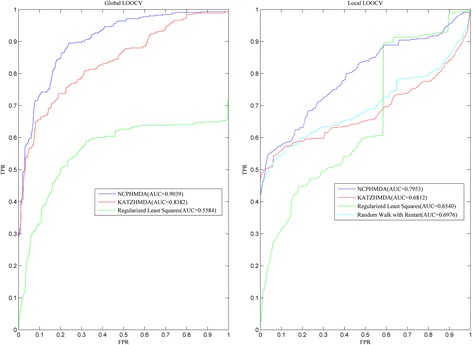
Performance comparisons between NCPHMDA and three state-of-art prediction models (KATZHMDA, Regularized Least Squares and Random Walk with Reastart) in terms of ROC curve and AUC. As a result, NCPHMDA achieved AUCs of 0.9039 and 0.7953 based on global and local LOOCV, significantly outperforming previous classification models

In this paper, we compared NCPHMDA with KATZHMDA [29], which has achieved excellent performance in potential microbe-disease association prediction. Two other previously proposed prediction methods (i.e. Regularized Least Squares [30] and Random Walk with Restart (RWR) [31]) were also applied to evaluate the prediction ability of NCPHMDA. To be clear, RWR algorithm only could predict associated microbes for given diseases and could not infer all the missing associations for all the diseases simultaneously. Therefore, global LOOCV couldn’t be implemented for RWR. In global LOOCV framework, NCPHMDA reached AUC of 0.9039 which had 0.0657, 0.3455 increase compared with KATZHMDA and Regularized Least Squares (See Fig. 2). In addition, NCPHMDA achieved AUC of 0.7953, which had 0.0977, 0.1141 and 0.1413 increase compared with Regularized Least Squares, KATZHMDA and Random Walk with Restart. Furthermore, 5-fold CV was also implemented. As a result, NCPHMDA yielded a reliable performance of 0.8918 +/− 0.0105. In conclusion, NCPHMDA has reliable performance in the framework of cross validations.

### Case studies

NCPHMDA was implemented to prioritize candidate microbes of all investigated diseases in this study. For further prediction ability evaluation, three kinds of complex human diseases (i.e. colon cancer, asthma and type 2 diabetes) were taken as three independent case studies. Based on recent published clinical and biological reports, predicted microbes ranked in top 10 of these three complex diseases were validated respectively. Importantly, it should be noted that only microbe-disease pairs without known evidence collected in HMDAD database were classed into validation datasets, which guaranteed the absolute independence between validation candidates and known associations used for model training.

According to the well-known global cancer statistics report [32], colon cancer occupied the third leading cause of cancers in males and the second leading cause of cancers in females in the past few decades. With the improved treatment and increased awareness, death rates of colon cancer patients have been decreasing in several developed countries. However, survival rates in developing countries are still far from meeting requirements because of the low detection rates in early stage. Recently, accumulating evidence have demonstrated that imbalance of microbial community has a close connection with occurrence and development of colon cancer. For example, Moore et al. [33] compared fecal floras of polyp patients (at high risk of colon cancer), Japanese-Hawaiians (at high risk), rural native Japanese (at low risk), rural native Africans (at low risk) and North American Caucasians (have a flora composition intermediate between two groups) and identified 15 colon cancer-related bacterial taxa. Surprisingly, they found that concentrations of *Bacteroides* and *Bifidobacterium* were positively related with colon cancer risks while concentrations of *Lactobacillus* and *Eubacterium aerofaciens* were negatively correlated with colon cancer risks. We implemented NCPHMDA on colon cancer for potential microbe-disease association prediction, and 9 out of the top 10 predicted microbes were successfully confirmed by biological literature (See Table 1). Typically, it is reported that colon cancer patients who have undergone preoperative insertion of a metallic stent and are aged sixty and older years are identified as risk factors for *Clostridium difficile* (1st in the prediction list) infection [34]. *Helicobacter pylori* (2nd in the prediction list) infection was found to be associated with risk increase of left-sided colorectal cancer [35]. By sequencing of 16S rRNA gene V3 region, abundance of *Proteobacteria* (3rd in the prediction list) was discovered under-represented in sporadic colorectal carcinoma patients [36].

**Table 1:** For further prediction performance evaluation, NCPHMDA was implemented on colon cancer to identify potential associated microbes. As a result, 9 out of the top 10 predicted microbes have been verified based on recent experimental literature

Rank	Microbe	Evidence
1	*Clostridium difficile*	PMID:21152135
2	*Helicobacter pylori*	PMID:22294430
3	*Proteobacteria*	PMID:25699023
4	*Prevotella*	PMID:25699024
5	*Staphylococcus aureus*	unconfirmed
6	*Clostridium coccoides*	PMID:18237311
7	*Firmicutes*	PMID:25699024
8	*Bacteroidetes*	PMID:25699024
9	*Actinobacteria*	PMID:26811603
10	*Clostridia*	PMID:19807912

Asthma is a common chronic inflammatory disease of the airways of the lungs, which is generally believed to be caused by a combination of genetic and environmental factors [37]. Recent statistics indicated that incidence of asthma has been in the increasing trend in the past few decades, and the number of asthma patients grew from 183 million in 1990 to 242 million in 2013 [38]. Infection of pathogenic microorganisms (especially virus, chlamydia, mycoplasma and mold) is one of the leading causes of severe asthma. For example, Huang et al. [39] have discovered that differences in the bronchial airway microbial composition were correlated with the manifestation of clinical asthma features. They pointed out the direct link between abundance of *Sphingomonadaceae*, *Comamonadaceae*, *Oxalobacteraceae* and degree of bronchial hyperresponsiveness among asthmatic patients. By implementing NCPHMDA to prioritize candidate microbes, 9 out of the top 10 predicted microbes were successfully verified by recent clinical evidence (See Table 2). As for top 5 confirmed asthma-related microbes, concentrations of *Clostridium difficile* and *Staphylococcus aureus* (1st, 5th in the prediction list) were discovered increased in asthma patients’ airways, while concentrations of *Firmicutes* and *Actinobacteria* were found decreased [40–42]. Importantly, *Clostridium coccoides* (3rd in the prediction list) subcluster XIVa species were proved serving as early indicators of possible asthma later in life, which could help prevent and diagnose asthma and provide guidance for clinical treatment [43].

**Table 2:** We implemented NCPHMDA on asthma to prioritize candidate microbes. As a result, 9 out of the top 10 predicted microbes have been confirmed based on recent experimental literature

Rank	Microbe	Evidence
1	*Clostridium difficile*	PMID:25974301
2	*Firmicutes*	PMID:23265859
3	*Clostridium coccoides*	PMID:21477358
4	*Actinobacteria*	PMID:23265859
5	*Staphylococcus aureus*	PMID:12743582
6	*Lactobacillus*	PMID:20592920
7	*Clostridia*	PMID:21477358
8	*Burkholderia*	PMID:24451910
9	*Lachnospiraceae*	PMID:17433177
10	*Enterococcus*	unconfirmed

According to recent disease statistic reports [44], diabetes mellitus represents 8.3% of the adult population and occupies the eighth leading cause of deaths annually. Type 2 Diabetes Mellitus (T2DM) makes up approximate 90% of all diabetes mellitus cases and can lead to chronic complications including cardiovascular diseases, stroke and diabetic retinopathy. Increasing evidences have shown that formation and development of T2DM are closely related to low-grade inflammation and microbial infection [45]. Compositional changes in intestinal microbiota such as *Bacilli*, *Bacteroidetes*, *Betaproteobacteria*, *Clostridia*, *Clostridium*, *Firmicutes*, *Lactobacillus* and *Proteobacteria* were discovered in T2DM patient feces [46]. We took T2DM as a case study for potential T2DM-related microbe prediction, 8 out of the top 10 predicted microbes were confirmed by experimental reports (See Table 3). *Helicobacter pylori* (1st in the prediction list) infection was found to be involved in pathogenesis of insulin resistance in T2DM patients, which could be regarded as important biomarker for early detection of high blood glucose and prevention of high-risk T2DM communities [47]. Zhou et al. [48] attempted to investigate the potential effect of T2DM on subgingival plaque of periodontal patients, and the results indicated that the abundance of *Prevotella* (3rd in the prediction list) was significantly different between diabetics and non-diabetics in subjects with healthy periodontium while populations of *Actinobacteria* (4rd in the prediction list) were significantly different between diabetics and their non-diabetic counterparts in subjects with periodontitis. Evidence of dysregulation of *Clostridium difficile* and *Staphylococcus aureus* (2nd and 5th in the prediction list) could be concluded from these clinical reports [49, 50].

**Table 3:** NCPHMDA was implemented on type 2 diabetes to identify potential related microbes. As a result, 8 out of the top 10 predicted microbes have been confirmed based on recent experimental literature

Rank	Microbe	Evidence
1	*Helicobacter pylori*	PMID:24782613
2	*Clostridium difficile*	PMID:23734349
3	*Prevotella*	PMID:23613868
4	*Actinobacteria*	PMID:23613868
5	*Staphylococcus aureus*	PMID:16495627
6	*Lachnospiraceae*	unconfirmed
7	*Staphylococcus*	PMID:24385898
8	*Haemophilus*	unconfirmed
9	*Bacteroides*	PMID:20140211
10	*Enterobacteriaceae*	PMID:25759592

Case studies on above three complex human diseases have confirmed the outstanding prediction ability of NCPHMDA. For further biological and clinical experiment validation, we prioritized and publicly released the prediction of all the unknown microbe-disease pairs (See Additional file 1). It is anticipated that the candidate microbe-disease pairs with higher ranks could offer valuable clues and would be confirmed by experimental observation in the near future.

## Conclusions

With the rapid development of high-throughput sequencing techniques, increasing literature have demonstrated that imbalance of microbial community has critical impacts on host’s health and disease. Identifying potential microbes associated with investigated disease for better understanding of disease pathology and novel discovery of drugs has attracted more and more attention in recent years. However, few computational models have been developed for potential microbe-disease association prediction, which could significantly reduce experimental time and cost that traditional clinical researches suffer. In this study, based on the assumption that microbes with similar functions tend to share similar association or non-association patterns with similar diseases, we presented a novel computational model named NCPHMDA to prioritize candidate microbe-disease pairs for further experiment validation. NCPHMDA achieved outstanding AUCs of 0.9039, 0.7953 and average AUC of 0.8918 in global LOOCV, local LOOCV and 5-fold CV, respectively. In addition, case studies of colon cancer, asthma and type 2 diabetes mellitus were implemented for further prediction ability evaluation. As a result, 9, 9 and 8 out of the top 10 predicted microbes of these three complex diseases were confirmed by recent literature evidence. It is anticipated that NCPHMDA could serve as an important resource providing essential supports for further clinical or biological researches.

In conclusion, the following factors drove the excellent prediction performance of NCPHMDA. First of all, known microbe-disease associations collected in HMDAD database are reliable as a basic information resource. Furthermore, Gaussian interaction profile kernel similarity for microbe and disease were integrated in NCPHMDA, which effectively improved the data completeness and further reduced model prediction bias. NCPHMDA could be implemented on new microbes without any known associated diseases as well as new diseases without any known associated microbes. In addition, NCPHMDA is a global ranking computational method and could prioritize all the candidate microbe-disease pairs for all investigated diseases in a large-scale.

It should be noted that some limitations still exist in the model design of NCPHMDA. Firstly, microbe-disease association network is sparse, which would limit the prediction accuracy of proposed model. This problem could be solved with collection of high-quality experimental microbe-disease associations in the future. Moreover, since calculation of Gaussian interaction profile kernel similarity was strongly relied on the known microbe-disease associations, the diseases with more known associated microbes are possibly predicted to be related with more potential microbes. Integrating more biological heterogeneous networks, such as disease phenotypic similarity network, disease semantic similarity network and microbe functional similarity network, could help improved the quality of existing networks and prediction performance of NCPHMDA. Establishing new similarity measures without dependence on the topological features of known microbe-disease association network is another improving direction which should never be ignored.

## Additional file

Additional file 1: Table S1. Prediction of all the unknown microbe-disease pairs. (XLSX 232 kb)
